# Perspectives, Challenges, and Advances in Therapeutic Strategies for Gynecological Malignant Tumors

**DOI:** 10.3390/biomedicines13020528

**Published:** 2025-02-19

**Authors:** Alberto Farolfi, Daniela Montanari, Chiara Casadei, Antonino Musolino

**Affiliations:** Medical Oncology, Breast & GYN Unit, IRCCS Istituto Romagnolo per lo Studio Dei Tumori (IRST) “Dino Amadori”, 47014 Meldola, Forlì-Cesena, Italy; daniela.montanari@irst.emr.it (D.M.); antonino.musolino@irst.emr.it (A.M.)

For years, treatment options for advanced gynecological malignancies have been limited, with the combination of carboplatin and paclitaxel being the preferred first-line therapeutic approach, regardless of disease type. However, given the better understanding in the pathogenetic mechanisms underlying the cancerogenesis of gynecological tumors and deeper genomic characterization, the therapeutic paradigm has changed radically in both cervical, endometrial, and ovarian cancer.

HPV infection has long been known to cause cervical tumors, but only recently has immunotherapy been shown to be effective, either alone [1] or in combination with chemotherapy for the treatment of recurrent or metastatic cervical cancer [2]. It is, therefore, not surprising that immunotherapy was tested in the early stages in combination with chemo-radiotherapy. Surprisingly, the results were conflicting, with the first study failing to meet its primary endpoint [3] and the second showing both improved disease-free survival (DFS) and overall survival (OS) [4]. Therefore, although the hypothesis that viral etiology may determine a response to immune checkpoint inhibitors (ICIs), the results emerging from randomized clinical trials have demonstrated that the biology underlying these diseases is more complex and needs to be better characterized. Similarly, neoadjuvant chemotherapy given prior to chemoradiation has been shown to improve the outcome of locally advanced cervical cancer [5]. In this context, it becomes necessary to research predictive biomarkers of response to immunotherapy and prognostic factors that may help to better identify the patient at risk of relapse.

No other treatment approach for a gynecological malignancy has changed as dramatically as the treatment of endometrial cancer, since genomic characterization was incorporated into daily clinical practice. The Cancer Genome Atlas (TCGA) classification grouped endometrial cancer into four distinct diseases, with not only prognostication capabilities but also predictivity for adjuvant treatments [6]. For instance, the POLE-mutated subgroup does not need any adjuvant treatment, whereas the p53 abnormal subgroup needs to be evaluated for adjuvant chemotherapy. Similarly to what happened to early-stage endometrial cancer patients with the integration of TGCA classification, the treatment paradigm for advanced endometrial cancer has changed to a more personalized approach [7]. Approximately 30% of advanced endometrial cancers are mismatch repair deficient or microsatellite instability high (dMMR/MSI-high). For these cases, the addition of ICIs to platinum-based chemotherapy has emerged as a new standard of care given the benefit in terms of progression-free survival (PFS) and OS [8,9,10]. For the proficient MMR (pMMR) population, the combination of ICIs with chemotherapy showed gains in terms of PFS but with a lower magnitude compared to the dMMR/MHS-high counterpart. Again, the use of poly(ADP-ribose) polymerase inhibitors (PARPi) in combination with ICIs as maintenance treatment of advanced pMMR endometrial cancer has been shown to improve clinical outcome [11]. In contrast with the clinical development seen in ovarian cancer, the use of PARPi in endometrial cancer is lacking predictive biomarkers of benefit. In ovarian cancer trials, patients were addressed to PARPi maintenance in case of a clinical/radiological response to platinum salts, and they were further stratified according to homologous recombination deficiency (HRD) status. By contrast, no clinical factors nor molecular biomarkers were used to select or stratify patients in Ruby part 2 and in the DUO-E trials [11,12]. As a consequence, the broader and heterogeneous population of pMMR patients is a potential candidate for four-agents [11] treatment without predictive biomarkers, raising concerns due to potential clinical and financial toxicities.

It must also be considered that as immunotherapy moves to the front line, fewer therapeutic options are available for subsequent lines. It is, thus, necessary to identify mechanisms of immune resistance and new targets for novel therapies.

The introduction of PARPi in maintenance therapy, both in first-line therapy and after a platinum-sensitive relapse, changed radically the therapeutic landscape of ovarian cancer patients. As widely recognized, several studies have investigated the use of PARPi in these settings, either as monotherapy or in combination with bevacizumab. The choice of the type of maintenance therapy depends on various factors, with BRCA and HRD status being among the most important. It was demonstrated, indeed, that a gradient in terms of improved PFS exists from HRD-negative, to HRD-positive, to BRCA-mutated patients [13,14,15]. It is essential to emphasize the importance of determining BRCA and HRD status at the time of ovarian cancer diagnosis, as they are prognostic and with hereditary/familial implications [16]. The HRD test also has predictive value, since the combination of olaparib and bevacizumab is effective only in the HRD-positive population [17]. Nonetheless, HRD-negative tumors still exhibited benefits from treatment with niraparib [14]. This discrepancy may arise from several factors, including the sensitivity and false-negative rate of the tests used to determine HRD status, unraveling the complexity of HRD assessment [18].

In this context, there are still unmet needs, as these studies are not directly comparable due to differences in selected population, the lack of randomization between PARPi and PARPi plus bevacizumab in HRD-positive patients, and between PARPi and bevacizumab in HRD-negative patients. In this regard, some comparative studies are currently ongoing, such as Nirvana1 (NCT05183984), MITO 25 (NCT03462212), and AGO-OVAR28 (NCT05009082), aiming to answer these questions. Again, it is still a matter of debate as to which is the best HRD test, capable of predicting the efficacy of platinum salts and/or PARPi. The need to develop dynamic tests arises from the fact that current tests are not sensitive to changes in HRD induced by chemotherapy or the development of resistance mechanisms to PARPi and, therefore, do not provide a dynamic result [18].

Finally, we must define an effective treatment for patients who progress during PARPi treatment or become platinum resistant. In this setting, the use of antibody–drug conjugates (ADCs) becomes relevant, with the MIRASOL trial demonstrating benefits in PFS and OS in tumors with high folate receptor-alpha (FRα) expression through the use of mirvetuximab soravtansine compared to standard chemotherapy [19]. Ongoing studies are still investigating the use of this drug in relapsed patients, including those who had received PARPi but are platinum-sensitive (PICCOLO trial) [20], as well as maintenance therapy in association with bevacizumab (GLORIOSA trial) [21]. The results appear to be very promising, although accompanied by a new toxicity profile, particularly ocular toxicity, which should not be underestimated.

In conclusion, the emerging trend in the new therapeutic strategies for gynecological cancers emphasizes the importance of tailoring treatment according to the biomolecular, histological, and genetic characteristics of the tumor. While the management of gynecological cancers remains a formidable challenge, the development of novel therapies, innovative treatment approaches, and the continuous progress in research provide us with the tools to confront this battle.
